# Histological Studies on a Newly Isolated *Bacillus subtilis* D10 Protease in the Debridement of Burn Wound Eschars Using Mouse Model

**DOI:** 10.3390/pharmaceutics13070923

**Published:** 2021-06-22

**Authors:** Ibtesam Al-Dhuayan, Essam Kotb, Amany Alqosaibi, Amal Mahmoud

**Affiliations:** 1 Department of Biology, College of Science, Imam Abdulrahman Bin Faisal University, P.O. Box 1982, Dammam 31441, Saudi Arabia; ialdhuayan@iau.edu.sa (I.A.-D.); amgosaibi@iau.edu.sa (A.A.); amhsalem@iau.edu.sa (A.M.); 2 Basic & Applied Scientific Research Center, Imam Abdulrahman Bin Faisal University, P.O. Box 1982, Dammam 31441, Saudi Arabia

**Keywords:** skin burn, wound healing, protease, silver sulfadiazine, *Bacillus subtilis*, collagen fibers

## Abstract

Background: Proteases are among the most important industrial enzymes, playing a critical role in the physiological, biochemical, and regulatory processes of all living organisms. This study evaluated the histological effects of a *Bacillus subtilis* D10 protease in combination with the antibacterial ointment silver sulfadiazine (SSD) on the burned skin of mice. Materials and Methods: The bacterial proteolytic enzyme was produced and purified through DEAE-Sepharose CL-6B and Sephadex G-100 FF. The in vitro protease specificity was then determined. The dorsal skin of albino mice was burned with 80% HCl solution, then treated under three conditions: cold cream, SSD, and SSD combined with the tested protease. After 15 days of daily treatment, the mice were sacrificed and skin tissue samples were histopathologically examined using hematoxylin eosin, and Masson trichrome staining. Results: The D10 protease hydrolyzed the proteinaceous components of eschars (fibrin, normal collagen, and denatured collagen) in vitro. Mice skins treated with protease and SSD mixture showed promising results, with more rapid healing than the other treatments. This group regenerated epidermis and dermis with newly formed granulated follicles, fibroblasts and blood capillaries in the dermis, and collagen fibers in the hypodermis. Conclusions: These results suggest that the serine protease produced by *B. subtilis* D10 promotes wound healing of mice skin burnt with HCl and restores the normal architectural pattern in a shorter time than the standard treatments.

## 1. Introduction

Burns are a serious medical issue throughout the world. Severe burns can financially, psychologically, and physically affect patients [1], resulting in hypermetabolism, sepsis, and mortality [2,3]. The rapid treatment of burn wounds is essential to promote the healing process. Wound healing is a complicated process, involving replacing damaged tissue with healthy functional tissue. The wound healing process has been exhaustively investigated, with a key focus on reducing the scarring [4]. It includes several stages: hemostasis, inflammation, proliferation, and tissue remodeling [5].

A number of compounds have been investigated for the treatment of burn wounds, with various antibacterial topical applications, each having advantages and disadvantages. Topical antibacterial agents are essential for burn treatments, as they aid in preventing the wound sepsis. Despite their success in preventing invasive infections, topical treatments do not eradicate bacteria from the wound. The currently available topical agents vary with respect to their effectiveness in inhibiting infection, side effects, and impact on the skin regeneration [6].

One well-known antibacterial topical agent for burn wounds is SSD [7]. It was first introduced by Fox in 1967 and has become the most common topical agent for burn treatments [8]. According to the instructions of the management of Cipto Mangunkusumo hospital, it is imperative to treat second-degree burns using SSD ointment [9]. SSD is a sulfonamide that is not only effective in treating burns due to its strong bactericidal efficacy but also its absorption is less than 10%; therefore, renal toxicity has not been demonstrated in many cases [6]. However, the use of SSD for a long time may lead to many side effects, such as renal toxicity, leukopenia, resistance to cream SSD, and delayed wound healing [9]. In addition, SSD delayed the burn restoration process in many cases [10], necessitating the involvement of a potent burn-healer that could be combined with SSD for burn wound treatments.

Proteases are biotechnologically valuable enzymes that catalyze the hydrolysis of proteins into peptides and amino acids [11]. These biomolecules play a significant role in healing, including burnt skin, because they support the natural healing of skin ulcerations by effectively removing dead proteinaceous tissues [12]. The bromelain protease extracted from pineapple has shown promising results in the eschar debridement; however, its uncertain purity has been as issue [13]. Relatively recently, Valachova et al. [14] identified and characterized several proteases in *Lucilia sericata* medicinal maggots that are involved in maggot debridement therapy.

Microorganisms, especially fungi and bacteria, have become the most important sources of industrially developed proteolytic enzymes. The use of these source organisms is favored owing to their affordable maintenance. Moreover, genetic engineering enables the proteases to be efficiently produced [15]. In addition, many of them are more stable than those extracted from animals and plants [16]. *Bacillus* species in particular have been found to produce several extracellular proteases which contribute to the wound healing process. For example, sutilains from *Bacillus subtilis* is the most common enzymatic debriding agent in burn therapy. However, the repeated application of sutilains over a long time is required, which may increase the risk of wound sepsis [17]. Vibriolysin from the marine bacterium *Vibrio proteolyticus* was successfully applied in the hydrolysis of the proteinaceous components of eschars (fibrin, elastin and in particular, collagen) [18]. This work aimed to evaluate the healing activity of an isolated *Bacillus subtilis* D10 protease in combination with the hydrophilic antibacterial ointment, SSD, on burn wounds of mice skin.

## 2. Materials and Methods

### 2.1. Source of the Protease-Producing Bacterium

Soil samples were gathered from various locations of Saudi Arabia and plated onto casein agar (pH 9.4) involving (g/L distilled water): agar (15), peptone (5), casein (5), and yeast extract (1). Incubation was carried out for 24 h at 40 °C. Cleared halos around the bacterial colonies were taken as the indicator of protease activity. The positive colonies were then purified through quadrate streaking onto nutrient agar petridishes (pH 9.0) composed of (g/L distilled water): agar (15), peptone (5), sodium chloride (5), meat extract (1), and yeast extract (2). Recovered bacterial isolates were then preserved in 20% (*v/v*) glycerol at −80 °C. The most potent isolate was D10, which was obtained from the rhizosphere region of the *Cistanche tubulosa* plant, Dhahran, Saudi Arabia. It was identified via *16SrDNA* gene sequencing. BLAST analysis showed that it is a strain of *B. subtilis* as it displayed a similar identity to an existing genus and species in the GenBank database. The nucleotide sequence was registered in the GenBank Data Library under the accession number MK819973.1.

### 2.2. Protease Production, Assay, and Quantification

The potential proteolytic enzyme from the local isolate number D10 was produced using the basal medium described by Kotb [19]. The organism was grown for 56 h at 40 °C in a 50 mL solution (pH 9.0) comprising of (g/L distilled water): soybean powder (5), lactose sugar (10), K_2_HPO_4_ (0.5), KH_2_PO_4_ (1.5), CaCl_2_ (2), and MgSO_4_.7H_2_O (0.5). Protein quantification was measured directly at A_280_. Protease activity was evaluated by mixing 1 mL of the enzyme with 1 mL of 1.0% (*w/v*) casein dissolved in 0.1 M glycine–NaOH buffer, pH 10.0. The enzyme–substrate mixtures were then incubated at 45 °C for 30 min. The reaction was terminated by the addition of 2 mL of 10% (*w/v*) trichloroacetic acid stop solution. The A_280_ of the supernatant was measured and converted to L-tyrosine equivalents. One unit of protease activity (U) was defined as the quantity of protease releasing one micromole of L-tyrosine equivalents /min under the standard conditions of the assay.

### 2.3. Enzyme Purification

After the production of the tested protease under the aforementioned conditions, the bacterial culture supernatant was fractionated by applying 30–60% (NH_4_)_2_SO_4_ precipitation. The resulting proteins were collected by centrifugation for 20 min at a speed of 10,000× *g* and resuspended in 6 mL of 20 mM borate buffer (pH 8.5). The first elution of the crude enzyme was carried out through the DEAE-Sepharose CL-6B column (2.5 × 30 cm^2^) equilibrated with the typical buffer. The unbounded proteins were eluted at a rate of 1 mL/min with the same buffer with a linear rise in the ionic strength from 20 mM to 800 mM. Protease-active fractions were then concentrated and subjected to the second elution through Sephadex G-100 FF column (2 × 70 cm^2^) at a rate of 0.5 mL/min using 20 mM borate buffer (pH 7.4). The final potent protease fractions were lyophilized and analyzed for enzymatic purity by SDS-PAGE analysis according to the Andrews method [20] using 10% resolving gel and 4% stacking gel with a constant volt of 60 V.

### 2.4. In Vitro Protease Specificity

The in vitro fibrin hydrolysis was estimated by the incubation of bovine fibrin (10 mg/mL) at 37 °C in 0.2 M Tris-HCl buffer (pH 7.5) containing 1.0% physiological saline with stirring at 50 rpm. The tested enzyme was added to the reaction solution at 100 U/mL concentration. Samples were removed periodically and assayed for free amino acids with the ninhydrin method mentioned in Sigma-Aldrich protocols. For the determination of the exact mechanism of action against fibrin, the modified fibrin plate assay was applied at pH 7.8 as described by Kotb [21]. Wells of the plate were supplemented with the tested protease preparation at 0.5 µg concentration of the enzyme per well. Other wells of the same plate were supplemented with the tested enzyme mixed with 10 mU plasminogen per well. The plate was then incubated at 37 °C for 12 h.

Collagen hydrolysis was determined with the placental collagen as the reaction substrate. Reaction mixtures contained 10 mg of collagen per ml Tris-HCl buffer (pH 7.5) containing 1.0% physiological saline. The enzyme was added at a concentration of 100 U/mL at 37 °C. Stirring was applied at 50 rpm and the samples were removed periodically for analysis. The liberation of free amino acids and small peptides was determined by the ninhydrin assay as described previously. In parallel, the digestion of the denatured collagen was determined by the boiling of Achilles tendon collagen for 7 min before the assay. 

### 2.5. In Vivo Study

This study was approved by the Animal Ethical Committee of Imam Abdulrahman Bin Faisal University (Ethical institutional review board permit number: IRB-2018-10-105, 14 April 2018). Experimental animals were adult male albino BALB/C mice weighing 30–35 g (12 weeks old) obtained from the animal house at Imam Abdulrahman Bin Faisal University, Saudi Arabia. The animals were maintained at the animal house of the Institute for Researcher and Medical Consultations (IRMC, Dammam, Saudi Arabia) for 7 days prior to the experiments under the following conditions: stainless steel cages, 25–27 °C, humidity, 12 h day and night periods, and fed ad libitum.

Eighty percent (*v/v*) HCl was used to induce burnt surface areas on mice skin. Cold cream was prepared from the following ingredients: bee wax, sodium borate, mineral oil, paraffin wax, cetyl alcohol, and distilled water [22]. One percent (10 mg/g) SSD was obtained from Riyadh Pharma Medical and Cosmetic Products Co. Ltd. (Riyadh, Saudi Arabia) under license from Smith and Nephew Pharmaceuticals Ltd. (Hull, UK). The bacterial protease was prepared in the Research Units at the College of Science (Dammam, Saudi Arabia). 

### 2.6. Experimental Design

A total of eighteen mice were used and were randomly divided into three treatment groups (6 animals/group). In the control group (G1), animals were treated by topical application of the cold cream daily for 15 days. In the SSD-treated group (G2), animals were treated with 10 mg SSD/g body weight daily for 15 days, while in the protease and SSD-treated group (G3), animals were treated with 10 mg SSD and 3 kU protease/g body weight daily for 15 days.

### 2.7. Wound Creation 

A surface comprising approximately 2 cm^2^ dorsal skin (between the shoulders and top of the femur) of the mice was shaved using an electric shaver. This region was selected to prevent the animal from agitating the wound as the area is difficult for the mice to reach. The shaved surface was treated with 0.15 mL of 80% HCl to induce a chemical burn. After 24 h of treatment, the mice were maintained separately under sterile conditions in an isolated room cleaned with an antiseptic solution. To minimize the animals’ discomfort arising from the burning and treatment, the analgesic ketoprofen (0.15 mg/kg) was orally administered 24 h before burning of the skin and 3 days after the burn.

### 2.8. Group Treatments 

The burnt areas in G1, G2, and G3 were covered with cold cream, SSD (1% *w/w*) skin cream, and protease + SSD (1% *w/w*), respectively. After 15 d, the animals were anaesthetized by intraperitoneal injection of xylazine–ketamine (0.1 mL/10 g body weight) and the burnt skin samples were collected for histological and histochemical processing.

### 2.9. Histological Assays

The lesion biopsies of the skin were fixed in 10% neutral formalin for a minimum of one week. Microtome sections (5 μm thickness) were mounted on pre-coated slides before passing through the hematoxylin and eosin stain followed by alcohol dehydration and xylene clearing for the histological analysis [23] and Masson trichrome staining for the collagen fibers [24]. The processed sections were protected by coverslips and examined using an Olympus light microscope (Olympus Optical Co., Tokyo, Japan) fitted with a digital camera. All waste from the experiments was disposed of in accordance with the international regulations.

## 3. Results 

### 3.1. Enzyme Separation

**Figure 1:**
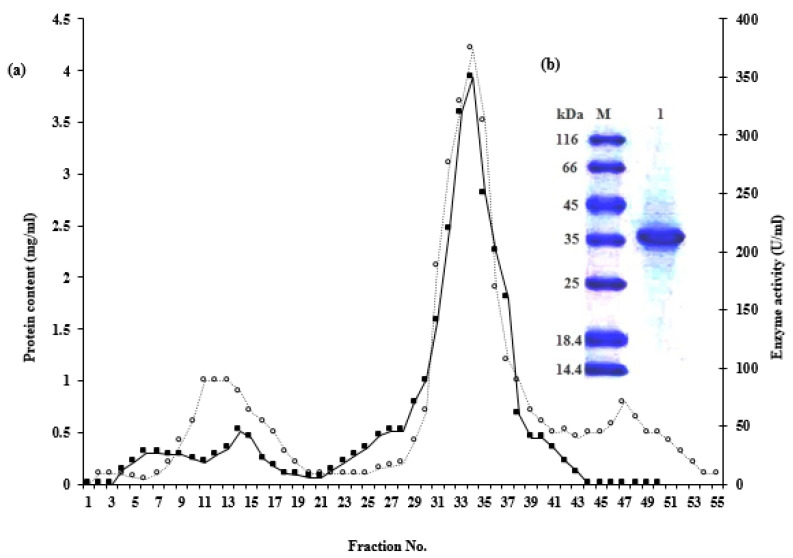
Elution pattern of protease through Sephadex G-100 (panel (**a**)) and SDS-PAGE (panel (**b**)). Lane M was loaded with the marker proteins, while lane 1 was loaded with the purified protease.

The bacterial isolate number D10 that showed the promising result in protease productivity was characterized molecularly as *B. subtilis* using *16SrDNA* gene fingerprint under the accession number MK819973.1. In addition, its serine protease gene was sequenced and given an accession number MK814957.1, and protein id QGA88714.1. Thereafter, three liters of the basal medium were prepared for enzyme productivity, and the protease was purified as briefed in Table 1. The last eluted protein on Sephadex G-100 (Figure 1a) showed a specific activity of 83.9-fold with 17.6% recovery and a major band at 37 kDa on SDS-PAGE (Figure 1b). Now, the purified protease preparation was ready for both in vitro and in vivo studies.

### 3.2. In Vitro Specificity of the Bacterial Protease

**Figure 2:**
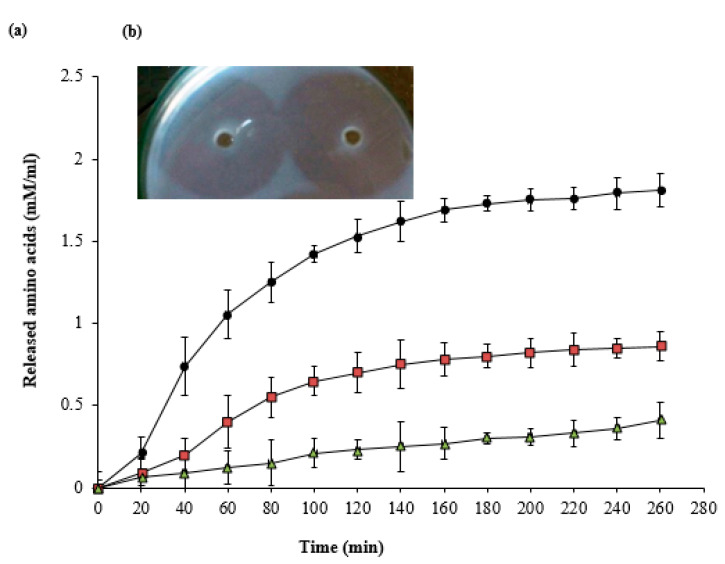
(**a**) Analysis of substrate specificity of the tested bacterial protease against the proteinaceous components of eschars (fibrin, native collagen, and denatured collagen). (**b**) Fibrin plate assay; left well contains 0.5 µg enzyme only per well (plasminogen-free well), but right well contains 0.5 µg enzyme and 10 mU plasminogen (plasminogen-rich well). The plate was incubated at 37 °C for 12 h.

According to Figure 2a, the tested enzyme hydrolyzed the proteinaceous components of eschars (fibrin, normal collagen, and denatured collagen) with various degrees. The digestion of denatured collagen was 2.10-fold in comparison with fibrin digestion and 4.41-fold in comparison with the digestion of normal collagen after 260 min treatment. The results of the fibrin plate assay (Figure 2b) showed that there was no difference in the diameter of the cleared halo around plasminogen-free and plasminogen-rich wells after 12 h of reaction.

### 3.3. Haematoxylin and Eosin Staining

**Figure 3:**
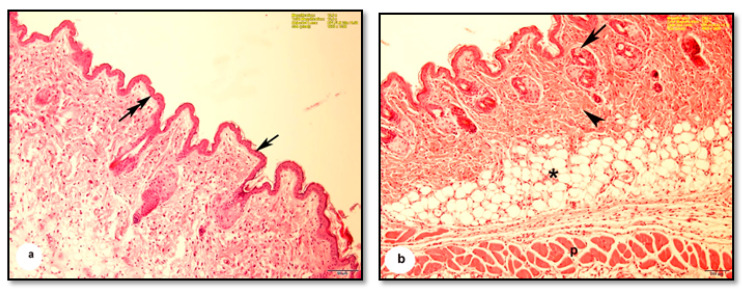
Photomicrographs of sections of normal mice skin before burning. (**a**) Showing the folded epidermis (arrow) based on the basement membrane (double-head arrow). (**b**) Showing the dermis (arrowhead) containing many hair follicles (arrow), bundles of fat cells forming adipose tissue arranged in lobules separated by septa of connective tissue (*), panniculus carnosus muscle (P) (H & E, scale bar: a = 100 µm, b = 100 µm).

The burn depth reached the deep dermis layer of treated mice, which is equivalent to the second to the third degree of burns. The histological architecture of normal mouse skin consists of two thin layers: the epidermis and dermis. The keratinized stratified squamous epithelial tissue of the epidermis is composed of two to three layers attached to the basement membrane (Figure 3a). The dermis consists of dense connective tissue containing numerous hair follicles. The hypodermis, or subcutaneous tissue, is located below the dermal layer and is comprised of loose connective tissue containing white fatty tissue arranged in lobules and separated by numerous septa of the connective tissue. Mouse skin is distinguished from human skin by the presence of the panniculus carnosus, which is a layer of skeletal muscle located beneath the dermal fatty tissue (Figure 3b).

**Figure 4:**
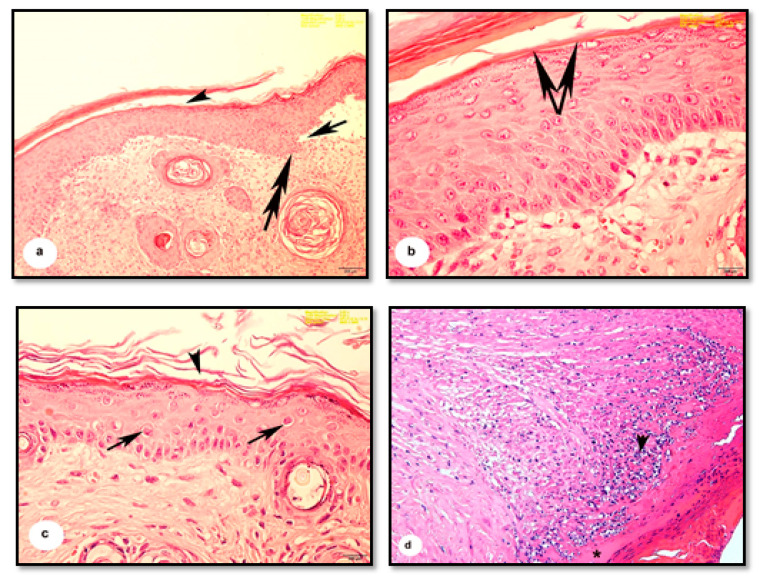
Photomicrographs of sections of burnt wounds treated by HCl 80% of the mice’s skin. (**a**) Showing distortion of the keratinized layer of the epidermis (arrowhead). Slight separation of the epidermis from the dermal layer at specific points (arrow) and discontinuous basement membrane (double-head arrow). (**b**) Showing thickening of the epidermis and termination of the horny layer from the epidermis (arrows). (**c**) Showing cytoplasmic degeneration of some epithelial cells (arrows). Severe disintegration and fragmentation of the horny layer (arrowhead). (**d**) Showing invasion of inflammatory cells in the dermal layer (arrowhead) and eosinophilic region (*). (H & E, scale bar: a = 100 µm, b = 400 µm, c = 400 µm, d: Magnification: 2.52×).

The dermis and epidermis were noticeably separated at specific points and were discontinuous with the basement membrane (Figure 4a). The horny layer of the epidermis was distorted and thickened, as was the epidermis (Figure 4b). Marked cytoplasmic degeneration was observed in some epithelial cells of the dermis (Figure 4c), which also showed cellular infiltrations and apparent eosinophilic tissues indicative of inflammatory changes (Figure 4d).

**Figure 5:**
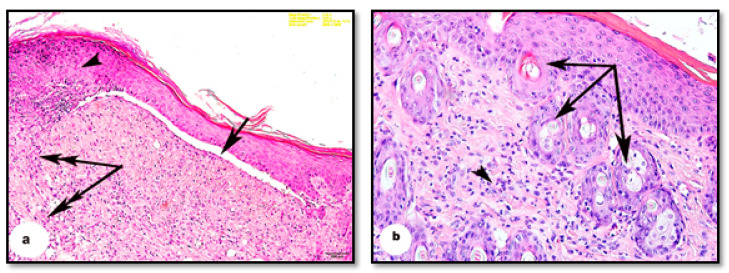
Photomicrographs of sections of burnt wounds in the mice skin treated with cold cream. (**a**) Showing thickening and distortion of the epidermal layer (arrowhead), irregularity of the Malpighian’s layer and its basement membrane (arrow). Additionally, typically reveals hyperkeratosis and follicular plugging, with superficial and deep lymphocytic, infiltrates (double arrows.). (**b**) Showing plugging of many epidermal follicles (arrows) and cellular infiltration formed granulation (arrowhead). (H & E, scale bar: a = 100 µm, b: Magnification: 2.52×).

**Figure 6:**
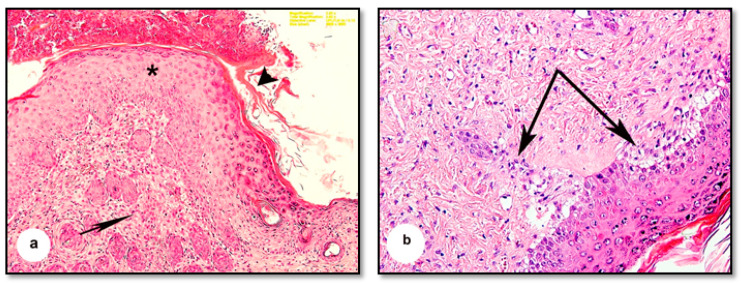
Photomicrographs of sections of burnt wounds in the mice skin treated with 1% SSD. (**a**) Showing highly thickened epidermal layer (*), some distortion of the horny layer (arrowhead.), moderate granulation of the dermis (arrow). (**b**) Showing slight cellular infiltration (arrows), (H & E, scale bar: a = 100 µm, b: Magnification: 2.52×).

**Figure 7:**
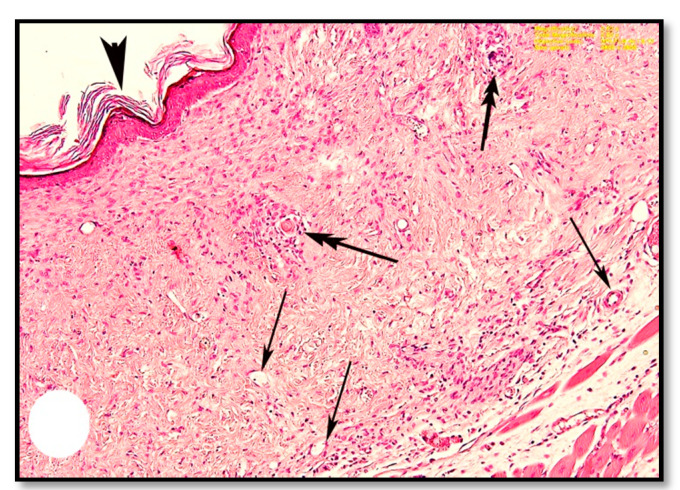
Photomicrographs of sections of burnt wounds in the mice skin treated with 1% SSD and protease enzyme. Showing nearly normal regenerated epidermis (arrowhead) and dermis with newly formed granulated follicles (double arrows) and rich with blood vessels (arrows). (H & E, Magnification: 2.52×).

The thickening and distortion of the epidermal layer were still apparent following treatment with cold cream (G1) or 1% SSD (G2). G1 mice displayed irregular Malpighian epithelial cells and basement membrane (Figure 5a). They also suffered from hyperkeratosis, apparent as follicular plugging and superficial and deep lymphocyte infiltration (Figure 5a,b). G2 mice displayed moderate granulation of new hair follicles in the dermis and only minimal cellular infiltration (Figure 6a,b). In contrast, following treatment with 1% SSD in combination with the *B. subtilis* D10 protease (G3), the histological architecture appeared nearly typical. The tissues showed complete wound healing, a regenerated epidermis and dermis with newly formed granulated follicles, and multiple dermal fibroblasts and blood capillaries (Figure 7).

### 3.4. Masson Trichrome Staining

**Figure 8:**
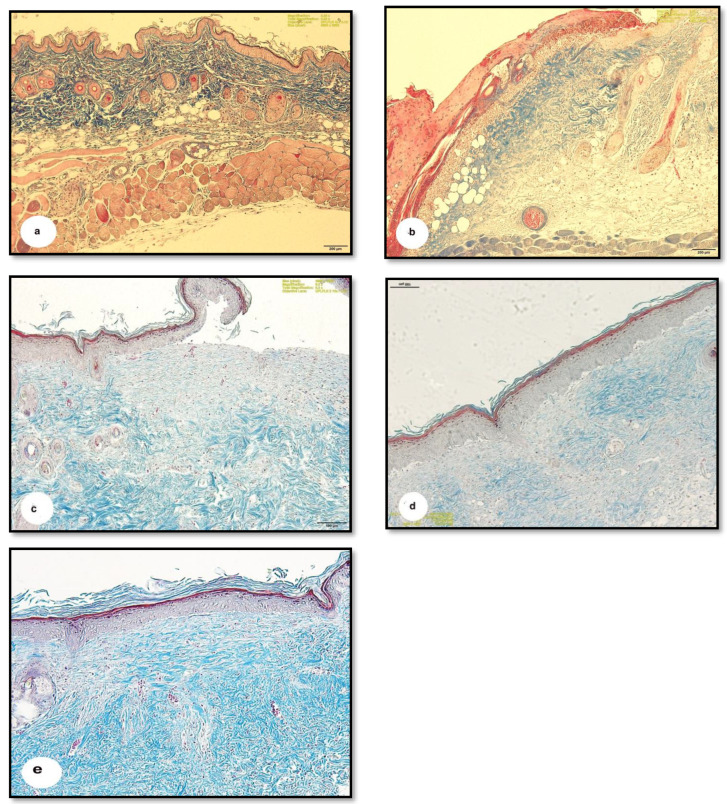
Photomicrographs of sections of the mice skin demonstrating the collagen fibers with blue color. (**a**) Showing the normal skin with thick and plenty of collagen fibers in the dermal region, (**b**) showing the burnt skin with thinner and fewer fibers, (**c**) showing the burnt skin treated with cold cream with moderate expression of the blue collagen fibers, (**d**) showing the burnt skin treated with 1% silver sulphadiazine demonstrating pale collagen fibers in the hypodermal region, and (**e**) showing the burnt skin treated with 1% silver sulphadiazine and protease enzyme revealing dens highly and darkly stained collagen fibers in the hypodermal region. (Masson trichrome, scale bars: a = 100 µm, b = 100 µm, c = 100 µm, d = 100, e: Magnification: 6.3×).

Masson trichrome staining was used to demonstrate the distribution of collagen fibers in the connective tissues of the skin. The hypodermal layer of normal skin was rich in dense collagen fibers (Figure 8a), while the HCl-burnt skin had thinner and less dense collagen fibers (Figure 8b). The moderate expression of blue-colored collagen fibers was apparent in the tissues of G1 mice (Figure 8c), and faint, pale-colored collagen fibers were visible in the skin of G2 mice (Figure 8d). On the other hand, G3 mice showed significant tissue improvement, with highly dense and darkly stained collagen fibers in the hypodermis (Figure 8e).

## 4. Discussion

Bacterial proteolytic enzymes, especially those produced by non-pathogenic bacteria, have a special concern in many medical and non-medical applications [25]. This study evaluated the potentiality of a serine protease from the non-pathogenic bacterium *B. subtilis* on the healing of burn wounds in mice. To determine the substrate specificity of our enzyme, an in vitro study was carried out with native collagen and denatured collagen in addition to fibrin as substrates. As shown in Figure 2a, the debriding enzyme exhibited superior proteolytic activity towards the denatured collagen over the native type ten times after about 4 h. The degradation of fibrin and collagen resulted in amino acids and short peptides that may induce wound closure and debridement in vivo at the gelatinized tissue and the necrotic cellular debris through keratinocyte migration and proliferation. Therefore, the purified enzyme offers the potential for rapid nonsurgical debridement of gelatinized burn tissue and the formation of new capillaries which will be noticed in the next in vivo study. This result is consistent with the sutilain protease ointment, which demonstrated a faster debridement in the hydrated wounds [26]. 

The mechanism of action of the tested enzyme against fibrin seems to be a direct degradation action (plasmin-like) and not a plasminogen activator since there was no difference in the diameter of the cleared halo of both plasminogen-free and plasminogen-rich wells after enzymatic reaction (Figure 2b). This ability may facilitate the removal of fibrin cuffs and components of the extracellular matrix inside the burns, opening the way and blood flow for the movement of cell precursors and the formation of new cells and connective tissue. These in vitro mechanistics were reflected during the next in vivo study by Masson trichome staining and hematoxylin eosin staining of burnt skin sections (Figure 7 and Figure 8e).

Figure S1 in Supplementary Material represents the wound closure trend due to the cold cream (b), silver sulfadiazine alone (c), and silver sulfadiazine and protease mixture (d) during the course of treatments. Burnt mice skin have undergone inflammatory changes, including cellular infiltrations in the dermis, in agreement with previous observations of rat skin burnt with a heated brass rod [27]. Previous studies have shown oedema, perfusion deficiency, and inflammatory infiltration to be common features of skin burns, regardless of the source of the wound [28,29,30]. A cleft formation between the epidermal and dermal layers has also been observed, with the initial separation between the basement membrane and epithelium occurring when the skin is exposed to temperatures of 200–300 °C [31]. We observed a similar separation in our study, along with a remarkable thickening of the epidermis and terminus of the horny layer in the skin. Scab formation and thickening of the epidermal layer have also been observed after burning mice skin with hot water (98 °C water, 10 s) [32]. 

Previous studies have controlled burns by preventing these phenomena [33,34]. One successful treatment involved the use of SSD, which is known to inhibit the growth of numerous microorganisms. However, it did not affect the inhibition of epithelial regeneration, nor did it prevent the autolysis of necrotic tissue by the proteolytic enzymes and collagenases [6]. Our findings showed improved wound healing in mice treated with 1% SSD; however, the healing process was incomplete after 15 d. This agrees with previous studies that found burn wound healing using SSD required prolonged treatment times [4].

In fact, wound healing is a complicated process that involves three integrated dynamic stages: inflammation, proliferation, and re-epithelialization. The inflammation stage restores homeostasis and integrity to the tissue. The proliferative phase begins when fibroblasts and other cells infiltrate the wound site. Fibroblasts secrete cytokines, which attract keratinocytes to the injury site, leading to re-epithelialization of the wound [35,36]. All the three stages were observed in the sections of mice skin treated with SSD and the tested serine protease, where the tissue showed complete wound healing, regenerated epidermis and dermis with newly formed granulated follicles, and numerous fibroblasts in the dermal tissue. The proteolytic enzyme likely promoted the natural healing process through the removal of necrotic debris, as suggested by Sjodahl et al. [37]. 

Blood capillaries were more apparent in the combined protease/SSD treated group. This aligns well with work from Apsari et al. [5], who established that vascular formation (angiogenesis) is part of structural restoration and is crucial for wound healing by supplying the epithelial cells and fibroblasts with oxygen and nutrients needed for tissue repair. McCarty and Percival [38] explained that the degeneration and remodeling of the extracellular matrix by proteases, particularly matrix metalloproteinases (MMPs), play a vital role in wound healing by promoting the influx of leukocytes, the formation of new blood vessels, and re-epithelialization.

Collagen fibers are essential to wound healing through the formation of fibers in the wound area providing strength to the tissue [5]. Darby et al. [39] illustrated that the proliferation phase of wound healing is distinguished by the presence of fibroblasts, which are involved in collagen fibre deposition. Collagen type I directs epithelial cell division and along with fibronectin plays a vital role in the healing process [40]. The strengthening of collagen fibers continues into the final stage of wound healing [36]. Kendall and Feghali-Bostwick [41] reported that fibroblasts are critical for re-epithelialization, the secretion of the extracellular matrix, and collagen production. Our study found a decrease in the density of collagen fibers in burnt skin as compared to the normal hypodermis, similar to previous studies indicating collagen denaturation in the skin of rats exposed to heated brass rods [27]. Treatment with SSD and protease resulted in the formation of highly dense collagen fibers in the hypodermis. Other studies have also shown the formation of highly dense collagen fibers, as well as fibroblasts, with wound healing treatments [32]. This study shows that treatment with 1% SSD coupled with protease improves wound healing as measured by fibroblast proliferation, inflammation, re-epithelialization, and neovascularization.

## 5. Conclusions

Our study confirmed that the serine protease produced by the locally isolated *B. subtilis* strain D10 has promoted wound healing when combined with SSD. This was through cell proliferation, inflammation, angiogenesis, and remodeling. This treatment speeds the recovery of burnt mice skin as compared to treatment with SSD alone. The selective nature of D10 debriding protease that was observed in the mouse model was a clear reflection of the intrinsic specificity of the enzyme as determined in vitro. This specificity is desirable in that it is selective for nonviable connective tissue; therefore, it does not damage the viable dermis. SSD is a hydrophilic cream that exerts its antibacterial action, and besides, can hydrate the eschar directly which allows more efficient diffusion of the debriding enzyme from the vehicle. 

Collectively, the in vitro and in vivo specificity of the tested protease to degrade the gelatinized proteins of burns is mostly the reason for opening the way and resuming the blood flow for the formation of new vessels at the burnt site (Figure 7). This has allowed the accumulation of fibroblasts at the injured site. Fibroblasts are known to secrete cytokines, that attract keratinocytes to the injury site, leading to the formation of highly dense collagen fibers in the hypodermis and re-epithelialization of the wound (Figure 8e). We, therefore, recommend the use of safe bacterial proteases in the preparation of topical treatments for burn wounds.

## Figures and Tables

**Table 1:** A brief of protease purification steps.

Purification Step	Total Activity (U)	Specific Activity (U/mg)	Purification Folds	Recovery (%)
Crude supernatant	513,122.5	39.9	1.0	100.0
Ammonium sulfate fractionation	221,362.3	832.5	20.9	43.1
DEAE-Sepharose CL-6B	145,325.0	3021.2	75.7	28.3
Sephadex G-100	90,258.2	3347.6	83.9	17.6

## Data Availability

Not applicable.

## Supplementary Materials

The following are available online at https://www.mdpi.com/article/10.3390/pharmaceutics13070923/s1, Figure S1. Wound closure trend due to the cold cream (b), silver sulfadiazine alone (c), and silver sulfadiazine and protease mixture (d). The status before the HCl burning is declared in panel (a).
