# Supplementary material for: Histological Studies on a Newly Isolated Bacillus subtilis D10 Protease in the Debridement of Burn Wound Eschars Using Mouse Model
Source: Pharmaceutics. 2021 Jun 22;13(7):923. doi: 10.3390/pharmaceutics13070923 (PMC8308810; doi:10.3390/pharmaceutics13070923)
Supplement: Supplementary file 1 [file pharmaceutics-13-00923-s001.zip › pharmaceutics-1223351-supplementary.pdf]

pharmaceutics

Supplementary Materials: Histological Studies on a Newly Isolated *Bacillus subtilis* D10 Protease in the Debridement of Burn Wound Eschars Using Mouse Model

Ibtesam Al-Dhuayan, Essam Kotb, Amany Alqosaibi and Amal Mahmoud

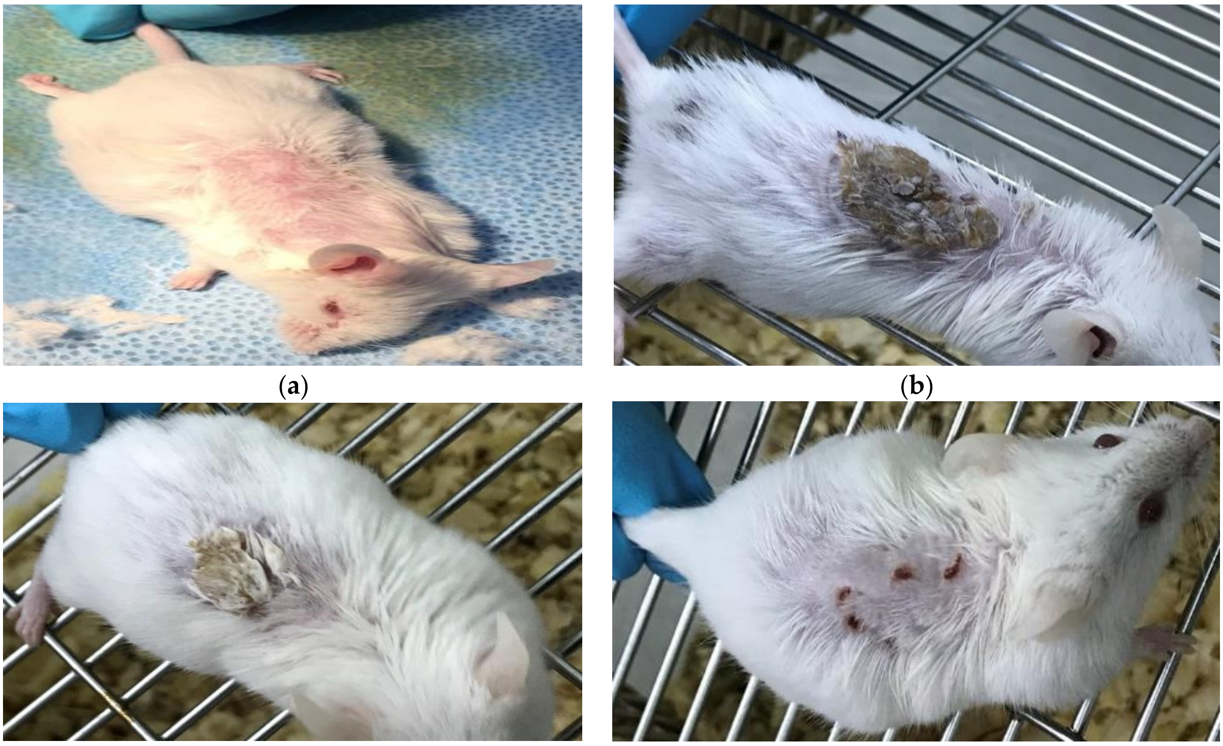

(**a**) (**b**) (**c**) (**d**)

Figure S1. Wound closure trend due to the cold cream (**b**), silver sulfadiazine alone (**c**), and silver sulfadiazine and protease mixture (**d**). The status before the HCl burning is declared in panel (**a**).
